# Comparative Genomics of Sibling Species of *Fonsecaea* Associated with Human Chromoblastomycosis

**DOI:** 10.3389/fmicb.2017.01924

**Published:** 2017-10-09

**Authors:** Vania A. Vicente, Vinícius A. Weiss, Amanda Bombassaro, Leandro F. Moreno, Flávia F. Costa, Roberto T. Raittz, Aniele C. Leão, Renata R. Gomes, Anamelia L. Bocca, Gheniffer Fornari, Raffael J. A. de Castro, Jiufeng Sun, Helisson Faoro, Michelle Z. Tadra-Sfeir, Valter Baura, Eduardo Balsanelli, Sandro R. Almeida, Suelen S. Dos Santos, Marcus de Melo Teixeira, Maria S. Soares Felipe, Mariana Machado Fidelis do Nascimento, Fabio O. Pedrosa, Maria B. Steffens, Derlene Attili-Angelis, Mohammad J. Najafzadeh, Flávio Queiroz-Telles, Emanuel M. Souza, Sybren De Hoog

**Affiliations:** ^1^Microbiology, Parasitology and Pathology Post-Graduation Program, Department of Basic Pathology, Federal University of Paraná, Curitiba, Brazil; ^2^Bioprocess Engineering and Biotechnology, Federal University of Paraná, Curitiba, Brazil; ^3^Laboratory of Bioinformatics, Sector of Technological and Professional Education, Federal University of Paraná, Curitiba, Brazil; ^4^Department of Biochemistry, Federal University of Paraná, Curitiba, Brazil; ^5^CBS-KNAW Fungal Biodiversity Centre, Utrecht, Netherlands; ^6^Institute for Biodiversity and Ecosystem Dynamics, University of Amsterdam, Amsterdam, Netherlands; ^7^Department of Cell Biology, University of Brasília, Brasilia, Brazil; ^8^Guangdong Provincial Institute of Public Health, Guangdong Provincial Center for Disease Control and Prevention, Guangzhou, China; ^9^Department of Clinical and Toxicological Analysis, Faculty of Pharmaceutical Sciences, University of São Paulo, Sao Paulo, Brazil; ^10^Pathogen and Microbiome Institute, Northern Arizona University, Flagstaff, AZ, United States; ^11^Department of Genomic Sciences and Biotechnology, Catholic University of Brasília, Brasilia, Brazil; ^12^Division of Microbial Resources (DRM/CPQBA), University of Campinas, Campinas, Brazil; ^13^Department of Parasitology and Mycology, School of Medicine, Mashhad University of Medical Sciences, Mashhad, Iran; ^14^Clinical Hospital of the Federal University of Paraná, Curitiba, Brazil

**Keywords:** *Fonsecaea* species, black yeast, genomics, chromoblastomycosis, comparative genomics, *Fonsecaea erecta*

## Abstract

*Fonsecaea* and *Cladophialophora* are genera of black yeast-like fungi harboring agents of a mutilating implantation disease in humans, along with strictly environmental species. The current hypothesis suggests that those species reside in somewhat adverse microhabitats, and pathogenic siblings share virulence factors enabling survival in mammal tissue after coincidental inoculation driven by pathogenic adaptation. A comparative genomic analysis of environmental and pathogenic siblings of *Fonsecaea* and *Cladophialophora* was undertaken, including *de novo* assembly of *F. erecta* from plant material. The genome size of *Fonsecaea* species varied between 33.39 and 35.23 Mb, and the core genomes of those species comprises almost 70% of the genes. Expansions of protein domains such as glyoxalases and peptidases suggested ability for pathogenicity in clinical agents, while the use of nitrogen and degradation of phenolic compounds was enriched in environmental species. The similarity of carbohydrate-active vs. protein-degrading enzymes associated with the occurrence of virulence factors suggested a general tolerance to extreme conditions, which might explain the opportunistic tendency of *Fonsecaea* sibling species. Virulence was tested in the *Galleria mellonella* model and immunological assays were performed in order to support this hypothesis. Larvae infected by environmental *F. erecta* had a lower survival. Fungal macrophage murine co-culture showed that *F. erecta* induced high levels of TNF-α contributing to macrophage activation that could increase the ability to control intracellular fungal growth although hyphal death were not observed, suggesting a higher level of extremotolerance of environmental species.

## Introduction

Melanized fungi belonging to the order Chaetothyriales are clinically relevant as agents of a gamut of diseases in humans and animals, varying in severity from superficial to systemic and fatal infections (De Hoog et al., 2004; Badali et al., 2008; Seyedmousavi et al., 2011). A large number of species have complex life cycles, indicating dynamic niches or vectored transmission (Sudhadham et al., 2008). In the environment, they occupy adverse micro-habitats, which is probably stimulated by their low competitive ability toward co-occurring microorganisms, judged from the fact that their isolation is enhanced significantly by the use of selective methods (Vicente et al., 2014). Many species of Chaetothyriales cause implantation diseases from an environmental source. One of the common disorders is chromoblastomycosis, a mutilating and recalcitrant skin disease eventually leading to emerging eruptions. Fungal cells in host tissue provoke as an inflammatory granulomatous disease (De Hoog et al., 2007; de Azevedo et al., 2015a; Queiroz-Telles, 2015). Agents of chromoblastomycosis are traumatically inoculated from environmental sources such as plant thorns or wooden splinters carrying the respective opportunist (Salgado et al., 2004; De Hoog et al., 2007; Vicente et al., 2014). Those species are mainly found in *Fonsecaea* and *Cladophialophora*, of which *F. pedrosoi, F. monophora*, and *Cladophialophora carrionii* are recurrently recovered from patients in tropical and semi-arid climate zones, respectively around the globe (Xi et al., 2009; Queiroz-Telles, 2015). Recently, less common agents were described, such as *F. nubica* (Najafzadeh et al., 2010), *F. pugnacius* (de Azevedo et al., 2015b), and *C. samoensis* (Badali et al., 2010). The genera *Fonsecaea* and *Cladophialophora* are morphologically classified by differences in their conidial apparatus; however, DNA polymorphisms suggest that both genera are polyphyletic (De Hoog et al., 2007), as they are distributed within “bantiana-clade” and “carrionii-clade” groups of the family Herpotrichiellaceae (Chaetothyriales) (Vicente et al., 2014; de Azevedo et al., 2015b).

Closely related species of *Fonsecaea* differ significantly in their ecology and ability to cause infection in humans and animals (Vicente et al., 2014); virulence genes seem to be unequally distributed among members of the bantiana-clade. *Fonsecaea pedrosoi* and *F. nubica* are strictly associated to chromoblastomycosis, while *F. monophora* also causes primary brain disease (Surash et al., 2005; Takei et al., 2007; Koo et al., 2010). *Fonsecaea multimorphosa* and *F. brasiliensis* were isolated from disseminated infections in animal hosts (Najafzadeh et al., 2011; Vicente et al., 2012) and the environmental species *F. erecta* and *F. minima* were described from plants and no report from clinical cases (Vicente et al., 2014) has as yet been published.

Therefore, the central question of the present study concerns the difference in infectious potential between closely related members of *Fonsecaea*. Agents of chromoblastomycosis upon tissue invasion show dimorphism to muriform cells, while this behavior is not known from most plant debris-inhabiting siblings (Queiroz-Telles, 2015). Comparative genomic analysis of *Fonsecaea* pathogenic and non-pathogenic siblings was applied to highlight genes involved in fungal adaptation from a plant debris- to an animal-associated life style. To this aim, a *de novo* assembly of *F. erecta* and recently published black yeast genomes (Bombassaro et al., 2016; Costa et al., 2016; Leão et al., 2017; Teixeira et al., 2017) was included in the analysis.

## Materials and methods

### Genomic DNA extraction, sequencing, and *de novo* assembly of *Fonsecaea erecta* CBS 125763^T^

A large-scale DNA extraction was conducted based on a method as described by Vicente et al. (2008). The strains were grown in Sabouraud broth for 7 days. The DNA was extracted by the cetyltrimethylammonium bromide (CTAB) method based and phenol-chloroform/isoamyl alcohol. Total DNA was purified with the Microbial DNA UltraClean™ kit. The genome was sequenced on MiSeq (Illumina™) sequencer using paired-end and mate-paired libraries and on Ion Proton (Thermo Fisher Scientific™) sequencer using single-end approaches. The library construction was done with Ion Plus Fragment Library Kit (Thermo Fisher Scientific™) and Nextera XT (Illumina™) following the manufacturer instructions. The read quality analysis was performed with FastQC and reads with quality below PHRED20 were removed (Andrews, 2016). The reads were assembled *de novo* using SPAdes v3.6.2 (Bankevich et al., 2012). The gap closure was performed with FGAP (Piro et al., 2014) and scaffolding with SSPACE (Boetzer et al., 2010). The mitochondrial genomes were assembled in *Fonsecaea* species by extracting reads using the complete mtDNA of *Exophiala dermatitidis* as reference. The reads were mapped using Bowtie2 (Langmead and Salzberg, 2012) and the mapped reads assembled with SPAdes v3.6.2 (Bankevich et al., 2012).

### Gene prediction and annotation

Protein-coding genes were predicted with GeneMark-ES v4.39 (Besemer, 2001). The automatic annotation was done by RAFTS3 (Vialle et al., 2016) best hits comparison with self-score cutoff of 0.5 using a black yeast protein database available on (www.broadinstitute.org/annotation/genome/Black_Yeasts/). Protein domain families and functional annotation was accessed using InterProScan5 (Quevillon et al., 2005). The tRNAs annotation used the ARAGORN software (Laslett and Canback, 2004). Putative enzymes and peptidases coding genes using CAZY (Cantarel et al., 2009) and peptidases coding genes using MEROPS database (Rawlings et al., 2015) and putative pathogen host interaction genes using PHI base (Winnenburg et al., 2007). Pathogen Host interacting (PHI) partners were identified by subjecting the predicted proteomes to BLASTp against the PHI database v4.2 with an *E*-value threshold of 10–5 with best hits.

### Comparative genomic analysis of the genus *Fonsecaea*

We compared the genome of *F. erecta* CBS 125763^T^ to 5 *Fonsecaea* species, including *F. monophora*, CBS 269.37^T^ (Bombassaro et al., 2016), *F. nubica* CBS 269.64^T^ (Costa et al., 2016), *F. multimorphosa* CBS 980.96^T^ (Leão et al., 2017), *Fonsecaea multimorphosa* CBS 102226 (Teixeira et al., 2017), and *F. pedrosoi* CBS 271.37^T^ (Teixeira et al., 2017) in addition to other 6 black yeast-like fungi belonging to the order Chaetothyriales (Teixeira et al., 2017): *Cladophialophora carrionii* CBS 160.54, *Cladophialophora yegresii, Capronia semiimmersa, Cladophialophora bantiana, Cladophialophora psammophila, Cladophialophora immunda*, and *Rhinocladiella mackenziei* (**Table 2**).

Protein sequences were compared using an all-vs.-all similarity search and self-score cutoff of 0.5 using RAFTS3 (Vialle et al., 2016). The clustering was done when at least one protein was shared amidst clusters. After the clustering verification step was done K-means and the cluster vectors where split into new clusters using the ratio of the cluster size and the number of organisms present in the analysis. For the resulting clusters it was calculated a centroid for each vector and chosen the best gene that represents each cluster based on the shortest distance. For both K-means and centroid analysis, a vectorial representation for the genes was created based on sparse *k*-mers sequences. A final clustering step was done using RAFTS all-vs.-all similarity searches with self-score of 90 (Vialle et al., 2016). The amino-acid sequences of each family were aligned with Muscle (Edgar, 2004) and poorly aligned regions were automatically removed using GBLOCKS (Talavera and Castresana, 2007). A maximum likelihood tree was done using PHYML (Guindon et al., 2005) and 1,000 bootstraps were used to infer branch support.

### Genome expansions and contractions based on functional domains

To identify functional expansions and contractions, InterPro domains were predicted using InterProScan5 (Quevillon et al., 2005) for 12 strains of black yeast species: 6 *Fonsecaea* species, 5 *Cladophialophora* species and 1 *Coniosporium apollinis* as outgroup (**Table 2**). Gene family evolution was estimated with CAFE version 3.0 (De Bie et al., 2006) using significance family-wide *p*-values threshold of <0.05 and VITERBI *p* < 0.001. To search for BIRTH (λ) values we run the program with the “-s” option. Two files were used as input in CAFE analyses: a table containing organism number of copies of each InterPro domain and an ultrametric tree.

### Prediction of genes related to pathogenicity

In order to research genes related to pathogenicity through analysis of the core, clusters and perform a correlation analysis, initially were rescued the 22 yeast genomes available at Broad Institute (http://www.broadinstitute.org/annotation/genome/Black_Yeasts/GenomesInde) and including the *Fonsecaea* sibling associated to (sub)cutaneous and systemic infection, totaling 26 genomes. They were all (re) annotated using toolbox RAFTS3 (Vialle et al., 2016). The next step was to vectorize and cluster each gene, which generated 28,355 gene clusters equal or bigger the 50% of identity between them. This analysis was sized in an array with 26 rows × 28,355 columns, the rows being the organisms and the columns all the genes of all clustered organisms.

To perform the correlation analysis between the clusters using Point-biserial correlation to each of 28,355 clusters, we selected a set of genes that was used as reference to access a set of already known pathogenic genes, being them: Cell Division Control Protein 42 (KIV82855.1), Cytochrome P450 (KIW97819.1), Thioredoxin (XP_013289847.1), HSP60-like protein (KIW92920.1), HSP90-like protein (AYO21_00238), Homogentisate 1,2-dioxygenase (KIW31930.1), and two hypothetical proteins (AYO21_05248 and KIW22607.1) which were present in the same clusters of paraoxonases.

**Figure 1:**
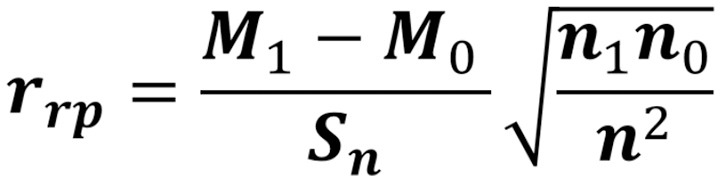
The point-biserial correlation coefficient: measure of the relationship between a continuous and a binary variable. For each protein of the 26 analyzed genomes, 0 and 1 scores correspond to the presence or absence of a protein of the binary variable, respectively. M1 is the mean of the presence of proteins and M0 is the mean of the missing proteins. The value “n” represents the total number of the proteins, where n1 is the total of proteins present and n0 are the total of the missing proteins. Sn is the standard deviation of the continuous variable.

The correlations of each cluster with the frequency of these pathogenic set genes in the organisms were calculated according to formula (Figure 1), which was filtered in 1,803 clusters of genes. Analyzing the gene families within organism related to systemic infection (*F. multimorphosa, C. bantiana, F. monophora*, and *R. mackenziei*) and subcutaneous infection (*F. pedrosoi, C. carrionii, F. monophora*, and *F. nubica*) only 280 showed positive correlation above 80% with 5.5 × 10^−7^ of max *e*-value.

### Virulence test of *Fonsecaea* sibling species using *Galleria mellonella* larvae as a model

#### Fungal strains, growth condition, and inoculum preparation

The strains *F. pedrosoi* ATCC 46428, *F. pedrosoi* CBS 271.37, *F. erecta* CBS 125763 and *F. monophora* CBS 102248 were selected for this study. The yeast strains were grown on Sabouraud Glucose Agar (SGA; Himedia, Mumbai, India) at 28°C for 7 days, transferred to Potato Dextrose Broth (PDB; Himedia) and incubated under agitation (150 rpm) at 37°C. After 5 days, the cultures were allowed to settle in order to decant the larger particles such as hyphae and conidia. Fungal cells were separated by filtration through a 40 μm cell strainer, (BD) washed with PBS 1 × three times. Finally, the cells were re-suspended at 1 × 10^6^ cells/mL for *F. pedrosoi* ATCC 46428, *F. pedrosoi* CBS 271.37, and *F. erecta* CBS 125763, or at 1 × 10^5^ cells/mL for *F. monophora* CBS 102248.

#### Larvae selection and infection

*Galleria mellonella* larvae were selected using as criteria similar size and weight ranging 0.10–0.15 (g). For the survival experiments, each group consisted of 20 larvae. The selected larvae were inoculated by injecting 10 μL of the different inoculum in the last left pro-leg with a Hamilton syringe (0.75 mm diameter needle) according to Fuchs et al. (2010). The control group was inoculated with PBS and the same number of larvae. The following control groups were used in the experiment. The first group included the larvae that received 10 μL of PBS to monitoring survival mortality related to trauma. A second group of larvae (SHAM) received no injection and no injury. All larvae were placed in sterile Petri dishes and kept in the dark at 37°C. Mortality was monitored once per day. The death of the larvae was assessed by the lack of movement, no response to stimulation and discoloration of the cuticle. Melanization was checked every 24 h with a NIKON D3100 camera and images were analyzed. Survival curves were plotted and statistical analyses were performed using the Log-rank (Mantel-Cox) test with Graph Pad Prism software. Statistical differences were set at *p* < 0.05.

#### Histopathology

Larvae were fixed by immersion in Carnoy (60% methanol, 30% chloroform and 10% acetic acid) 7 days after infection. After 48 h the larvae were immersed in 70% ethanol. Then the sections were then embedded in paraffin wax, sectioned and stained with Periodic Acid-Schiff (PAS) for microscopic examination. The photomicrographs were obtained from Olympus BX41 microscope coupled with digital camera Olympus SC30.

#### Fungal macrophage co-culture

The strains *F. pedrosoi* CBS 271.37 and *F. erecta* CBS 125763 were cultivated on Sabouraud Glucose Agar (SGA, Himedia) supplemented with 100 mg/L^−1^ chloramphenicol at 37°C. To obtain purified conidia and hyphae, fungi propagules were grown in PDB supplemented with 100 mg/L^−1^ chloramphenicol, in a rotary shaker (120 rpm) at 30°C. Fungal purification was performed according to Siqueira et al. (2017). Briefly, 15 days suspension containing conidia and hyphal fragments was subjected to successive filtrations through 70 μm and 40 μm cell strainers (BD). Hyphae retained on the 40 μm cell strainer were re-suspended in phosphate buffered saline (PBS), centrifuged at 1,000 × g and re-suspended in PBS. This process was repeated twice. Ninety-eight percent of 98% of this suspension consisted of hyphae. The 40 μm cell strainer filtrate containing conidia and small hyphal fragments was subjected to a filtration through 14 μm filter paper (J. Prolab, Brazil), washed twice in PBS, and recovered by centrifugation at 3,000 × g. This fraction contained conidia more than 98% pure.

Macrophage infection assays were adapted from Hayakawa et al. (2006), Palmeira et al. (2008) and Siqueira et al. (2017). Mouse macrophages (J774 cell line) were plated in DMEM (Dulbecco's Modified Eagle's medium, Sigma) supplemented with 10% heat-inactivated fetal bovine serum (Gibco) and infected with conidia or hyphae of the *Fonsecaea* species at a multiplicity of infection (MOI) of 1. After 3 h of infection, non-phagocytized fungi were washed, fresh medium was added and co-culture was allowed to proceed for 24 h. For fungal burden determination (number of fungal cells in macrophages), macrophages were lysed with 0.05% SDS solution. Intracellular fungi were quantified by plating serial dilutions of cell lysates onto SDA medium, supplemented with 100 mg/L^−1^ chloramphenicol and cultivated at 30°C for 7 days. Fungal burden was then measured by counting fungi colony-forming units (CFU). The cell culture supernatants were used to determine tumor necrosis factor-α (TNF-α) levels by enzyme-linked immunosorbent assay (ELISA), in accordance with manufacture's (eBioscience) instructions. As positive control to TNF-α production, 500 ng/mL LPS (*Escherichia coli* serotype 0111:B, Sigma-Aldrich) was used. Results were expressed as number of CFU or pg/mL of cytokine ± standard deviation (SD).

#### Mice infection

BALB/c mice were maintained under standard laboratory conditions. Mice (10–12 weeks old males) were inoculated by injecting 50 μL (per hind footpad) of PBS containing 1 × 10^6^ *F. pedrosoi* or *F. erecta* propagules obtained by mixing the purified hyphae and conidia in the proportion of 3:1, respectively. Five animals per group were euthanized with CO_2_ in an appropriate chamber at 7 and 14 days post-infection. The mice footpad were photographed, removed, weighed and thereafter homogenized in tubes with steel beads on a Precellys homogenizer. For fungal burden determination, homogenized tissue were diluted and plated as above mentioned. Results were expressed as number of CFU ± standard error of mean (SEM) per gram of fresh tissue. Cytokine production was measured from homogenized tissue obtained from infected and non-infected animals (healthy) by ELISA. The cytokines interleukin-1β (IL-1β), TNF-α, interleukin-6 (IL-6) and monocyte chemoattractant protein-1 (MCP-1/Ccl2) were measured with kits purchased from eBioscience and used according the manufacturer's instructions. Results were expressed as pg of cytokine ± standard error of mean (SEM) per 100 milligrams of tissue. All animal experimentation in this study were approved by the Federal University of Paraná Ethics Committee (approval certificate 1002) and performed according to the Committee's recommendations.

## Results

### *De novo* assembly of *Fonsecaea erecta* and genome contents of *Fonsecaea* siblings

Whole genome sequencing of *Fonsecaea erecta* CBS 125763^T^ was performed using Illumina Hiseq 2000 and yielded 1,534,038 paired-end reads with average insert size of 1 Kb ± 1 Kb and 3,133 mate-paired reads with average insert size of 5 Kb ± 4 Kb. To increase sequence coverage, two additional Ion Torrent shotgun libraries were sequenced generating 5.4 Gb and 25,219,375 single reads. The final high quality draft genome of *Fonsecaea erecta* comprised 57 scaffolds. The genome size was estimated to be 34.75 Mb, with average coverage of 60X and G+C content of 53%. Protein coding regions account for 18,279,031 bp, corresponding to 12,327 genes. A total of 12,090 proteins encoding genes, one rRNA multi-copy segment and 30 tRNA genes were predicted (Table 1).

**Table 1:** *Fonsecaea erecta* genome data assembly and quality.

**Information**	**Value**
Genome size (Mb)	34.75
DNA coding (bp)	18,279,031
DNA G+C (%)	53
DNA scaffolds	57
Coverage	60X
tRNA	30

Comparing *F. erecta* with related species of Chaetotryriales it was observed that all genomes used in this study including the reference species are similar in size. The genome size of *Fonsecaea* species varied between 33.39 and 35.23 Mb. The *F. monophora* genome is nearly 1.84 Mb larger in size following of the plant associate species *F. erecta* with 34.75, while the reference genome of the species plant-associated *Cladophialophora yegresii* presented a reduced size of 27.9 Mb. The total number of initial predicted genes in *Fonsecaea* varied between 11,681 in *F. nubica* to 12,527 in *F. pedrosoi*; between 10,944 (93.69%) and 11,948 (96.59%) of the genes identified as conserved hypothetical proteins. In addition, repetitive element identification was considered to be low in *Fonsecaea* siblings, ranging from 1.06 to 1.13% in *F. multimorphosa* to 1.93% in *F. monophora* (Table 2).

**Table 2:** Genome studied.

**Species**	**Strains**	**Source**	**Geography**	**GeneBank genome**	**Genome size (Mb)**	**G+C content (%)**	**Repetitive elements**	**Hypothetical proteins**
*Capronia semiimmersa*	CBS 27337	Human chromoblastomycosis	Brazil	JYCC00000000.1	31.62	53.7	0.71	92,23
*Cladophialophora bantiana*	CBS 173.52	Human chromoblastomycosis	USA	JYBT00000000.1	36.72	51.3	4.47	93,10
*Cladophialophora carrionii*	CBS 160.54	Human chromoblastomycosis	Australia	PRJNA185784	28.99	54.3	1.15	91,98
*Cladophialophora immunda*	CBS 834.96	Human skin lesion	USA	JYBZ00000000.1	43.03	52.8	2.24	93,00
*Cladophialophora psammophila*	CBS 110553	Gasolin-polluted soil	Netherlands	AMGX00000000.1	39.42	50.6	6.92	82,89
*Cladophialophora yegresii*	CBS 114405	*Stenocereus griseus* cactus	Venezuela	AMGW00000000.1	27.90	54	1.13	79,56
*Coniosporium apollinis*	CBS 100218	Pentelic marble	Greece	AJKL00000000.1	28.65	52.1	–	85,72
*Fonsecaea erecta*	CBS 125763	Living plant	Brazil	LVYI00000000.1	34.75	53	1.74	90,54
*Fonsecaea monophora*	CBS 269.37	Human chromoblastomycosis	South America	LVKK00000000.1	35.23	52.22	1.93	93,88
*Fonsecaea multimorphosa*	CBS 980.96	Cat brain abcess	Australia	LVCI00000000.1	33.39	52.64	1.06	96,34
*Fonsecaea multimorphosa*	CBS 102226	Decaying trunk palm tree	Brazil	PRJNA233317	33.45	52.6	1.13	96,59
*Fonsecaea nubica*	CBS 269.64	Human chromoblastomycosis	Cameroon	LVCJ00000000.1	33.79	52.46	1.59	93,69
*Fonsecaea pedrosoi*	ATCC 46428	Human chromoblastomycosis	South America	PRJNA233314	34.69	52.4	1.5	92,87
*Fonsecaea pedrosoi*	CBS 271.37	Human chromoblastomycosis	South America	PRJNA233314	34.69	52.4	1.5	92,87
*Rhinocladiella mackenziei*	CBS 650.93	Human cerebral phaeohyphomycosis	Saudi Arabia	JYBU00000000.1	32.47	50.4	3.49	92,48

### Mitochondrial genomes

**Figure 2:**
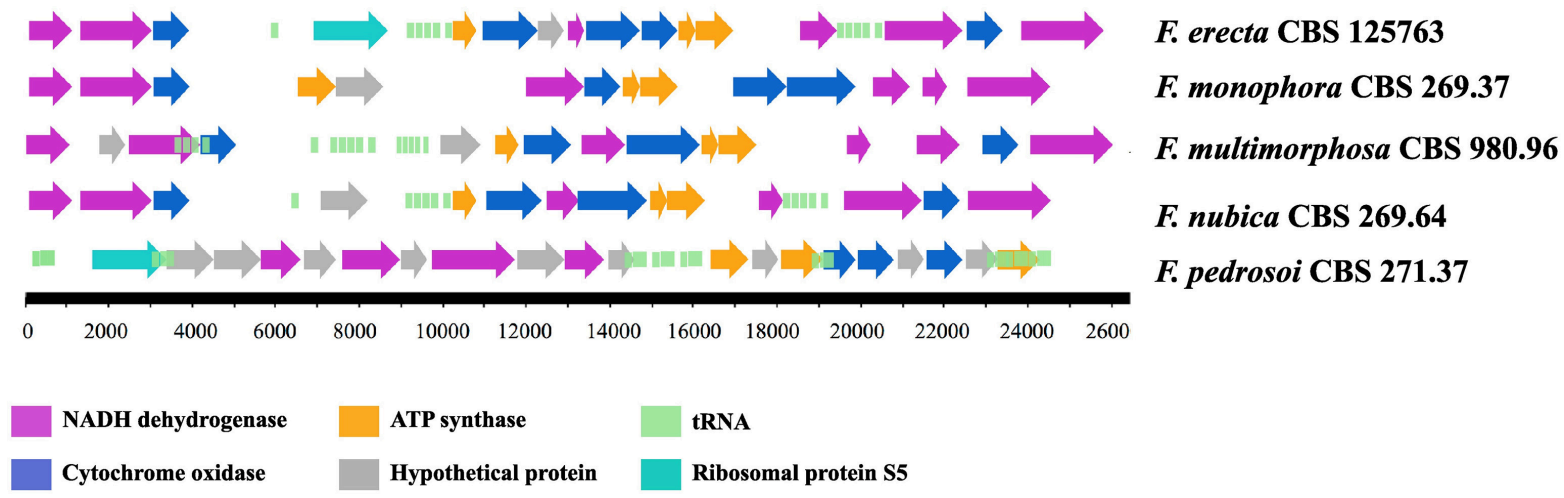
Mitochondrial genomes of *Fonsecaea* sibling species.

*Fonsecaea erecta* CBS 125763^T^ and *F. pedrosoi* CBS 271.37^T^ mtDNA was assembled in one contig, measuring 25.7 and 25 Kb, respectively. The mtDNA of *F. monophora* assembled into eight contigs comprising 24.7 Kb, the mtDNA of *F. nubica* resulted in a single contig with 24.5 Kb and the mtDNA of *F. multimorphosa* CBS 980.96^T^ resulted in seven contigs with a size of 26.4 Kb. Although the gene composition of all mitochondrial genomes analyzed shared 16 protein coding genes involved in the respiratory chain and ATP synthesis, the synteny of these genes is not conserved, showing rearrangements when compared between *Fonsecaea* species and with the reference sequence of *E. dermatitidis* (Figure 2).

### Orthologous gene comparison among *Fonsecaea* siblings

**Figure 3:**
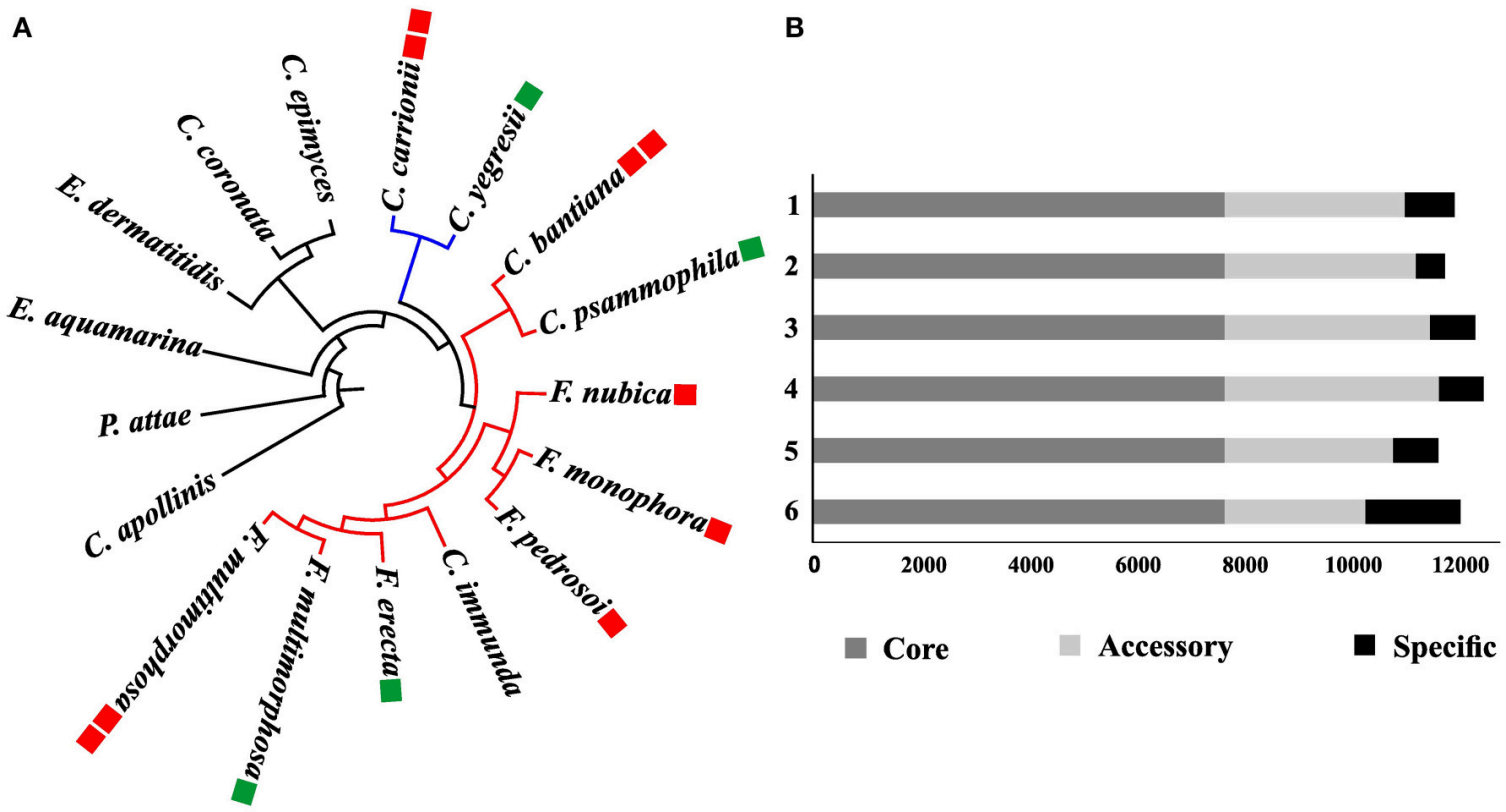
Phylogenomic analysis among *Fonsecaea* sibling species. **(A)** Phylogenetic tree based on the orthologous gene clusters. The bantiana-clade is indicated by a red line and the carrionii-clade by a blue line. Species names in green boxes are environmental strains, in red boxes agents of chromoblastomycosis and cerebral infection. **(B)** Gene cluster distribution in *Fonsecaea* siblings. Clusters of orthologous genes are showed in colors: dark gray core genes; light gray accessory genes, black specific genes. 1. *F. monophora* CBS 269.37, 2. *F. multimorphosa* CBS 980.96. 3. *F. multimorphosa* CBS 102226, 4. *F. pedrosoi* CBS 271.37, 5. *F. nubica* CBS 269.64, 6. *F. erecta* CBS 125763.

In order to study gene family evolution in *Fonsecaea*, we identified 20,713 orthologous gene clusters across 17 fungal genomes of environmental species and agents of subcutaneous and disseminated infection in animals and humans, including 9 species from the bantiana-clade and 2 from the carrionii-clade using *Coniosporium apollinis, Exophiala aquamarina*, and *Phialophora attae* as outgroup. The comparison showed a total of 5,727 of gene family clusters shared among *Fonsecaea* species, which was used to build a phylogenomic tree (Figure 3A).

The analysis showed strong support for published phylogenetic relationships between bantiana-and carrionii-clades in the order Chaetothyriales. Agents of chromoblastomycosis were distributed in the separate clades, with species associated with vertebrate infection clearly being distinct from environmental siblings. *Fonsecaea pedrosoi* and *F. nubica* causing chromoblastomycosis were closely affiliated with *F. monophora* involved in the same disease and as well as in brain infection. The plant-associated *F. erecta* is distinct from the clinical species. In addition, *F. multimorphosa*, an environmental species that once caused a feline cerebral abscess, formed a separate cluster with an environmental sibling (Figure 3A).

Cluster analysis was performed in the protein set of four *Fonsecaea* species: *F. erecta* CBS 125763^T^, *F. multimorphosa* CBS 980.96^T^ (Leão et al., 2017), *F. monophora* CBS 269.37^T^ (Bombassaro et al., 2016) and *F. nubica* CBS 269.64^T^ (Costa et al., 2016). The core genome comprised almost 70% (~8,000) of the genes (Table S1); the number of accessory genes (Table S2) in *F. erecta* was less than 20%, with specific genes (Table S3) lower than 10% (~2,000) (Figure 3B).

**Figure 4:**
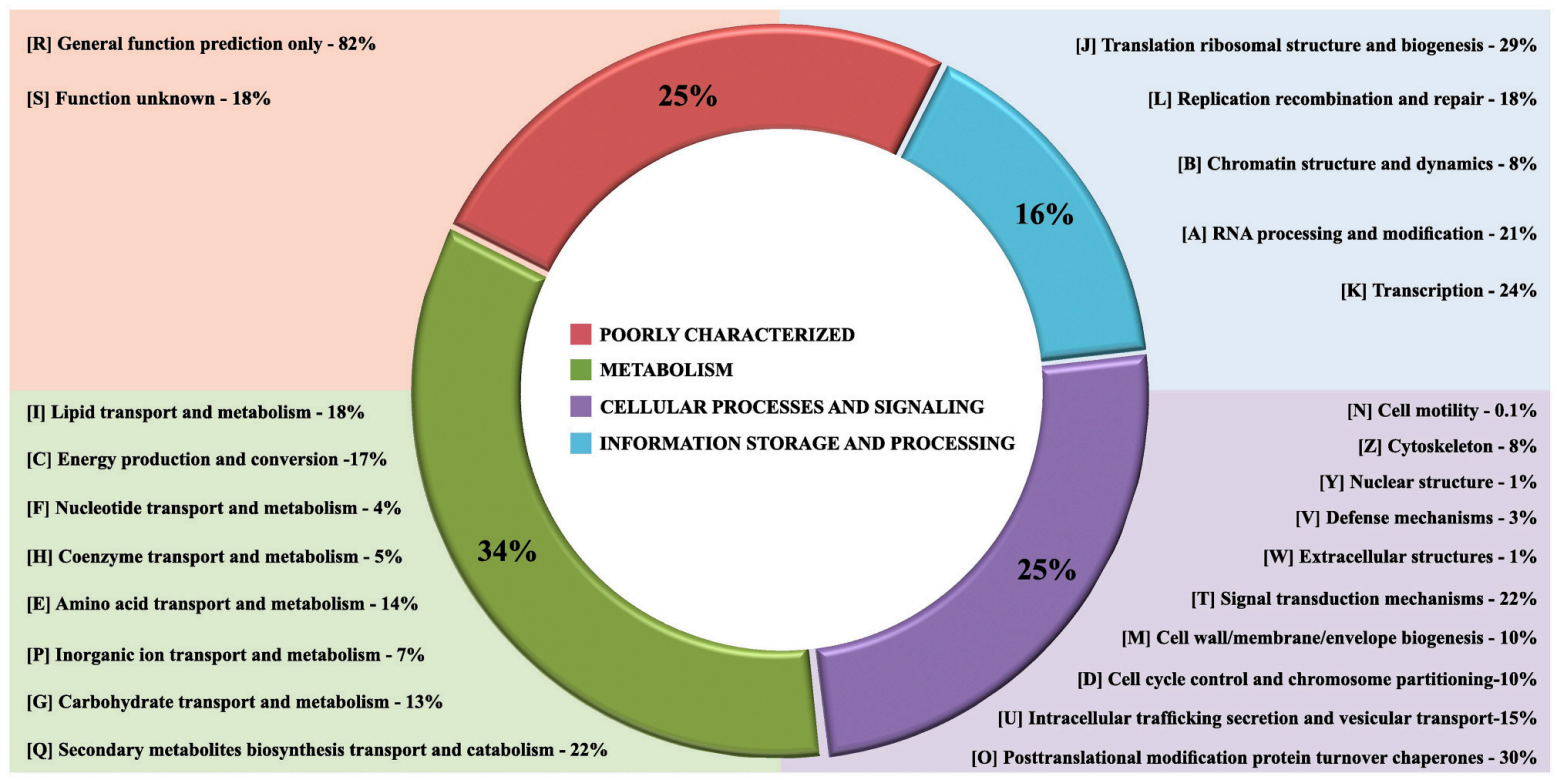
The core gene annotation from Eukaryotic Orthologous Group (KOG) in *Fonsecaea* species. KOG annotation by categories: In red poorly characterized proteins; in green metabolism category, in purple cellular processes and signaling category and processing and in blue category of information storage.

**Figure 5:**
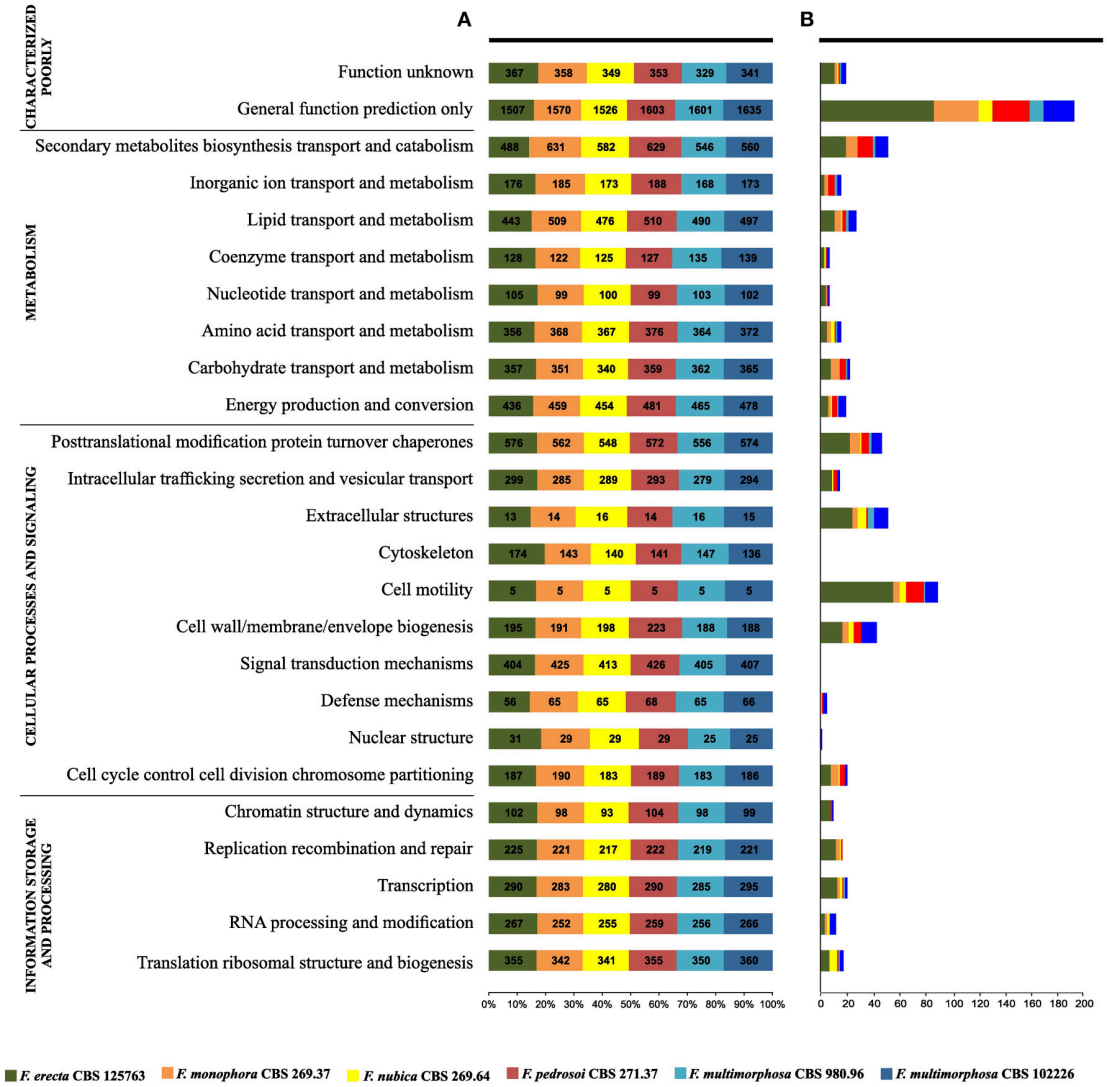
Gene families shared between *Fonsecaea* siblings based on annotation from Eukaryotic Orthologous Group (KOG). **(A)** The siblings are summarized in colored boxes. **(B)** Specific gene annotation among *Fonsecaea* siblings.

The gene core set was annotated with Eukaryotic Orthologous Group (Table S1). A total of 6,775 genes families clusters were redundantly assigned into KOG classifications, of which 2,259 genes were annotated as poorly characterized proteins, 1,382 assignments were in the category of Information Storage and Processing, 2,128 in the Cellular Processes and Signaling category and 3,265 in the Metabolism category (Figure 4). Of the eight KOG sub-classifications allocated in the Metabolism category, 718 genes were annotated to the Secondary Metabolites Biosynthesis, Transport and Catabolism [Q], 566 genes annotated to Lipid Transport and Metabolism [I] and 552 genes annotated to Energy Production and Conversion [C], composing the three more relevant classifications in this category. Moreover, in the Cellular Process and Signaling category, 631 genes were annotated with 51% proteins distributed in the Post-translational Modification Protein Turnover Chaperones class [O] with 450 genes annotated and the class Signal Transduction Mechanisms [T] and 326 genes in the Intracellular Trafficking Secretion and Vesicular Transport [U], composing the top three classifications in this category to each species analyzed (Figures 5A,B).

In the Q class (Figure 4) significant domains were identified as being common among *Fonsecaea* clinical and plant-associated species. The catalytic domain related to zinc-containing alcohol dehydrogenase (ADH) (IPR002328, IPR013149, IPR013154) is the most abundant domain in the *Fonsecaea* siblings which has been implicated in many biochemical pathways with a role in pathogenicity, stress and intoxication and were previously reported to be expanded in black yeast species (Das and Vasudevan, 2007; Grahl et al., 2011; Strommer, 2011; Teixeira et al., 2017). In addition, flavin-containing monooxygenases (FMOs) (IPR000960) constitute a family of xenobiotic-metabolizing enzymes that are widely present in *Fonsecaea* species. Moreover, related to metabolic processes, some protein domains in Q class were related to the biosynthesis of melanin and were widely found among *Fonsecaea* siblings: IPR011141, IPR019587, and IPR004235 with a role in DHN-melanin pathway, IPR002227 related to DOPA-melanin pathway, and IPR005708, IPR005955, and IPR005956 related to L-tyrosine degradation pathway. Some key enzymes involved in melanin biosynthesis were observed, including the family multicopper oxidases (MCOs) (IPR011706, IPR011707, and IPR001117). This family includes laccases, ferroxidases, bilirubin oxidases and ascorbate oxidases (Hoegger et al., 2006). Likewise, the superfamily aldehyde dehydrogenase (ALDH) (IPR015590, IPR008274) represents a divergently related group of enzymes that metabolize a wide variety of endogenous and exogenous aldehydes (Vasiliou et al., 2000).

In the T class (Figure 4) a caspase-like domain (IPR029030) and peptidase C14A, caspase non-catalytic subunit p10 (IPR002138), which are broadly classified as cysteine peptidases, were identified among the *Fonsecaea* species studied. Cysteine-dependent aspartyl-specific protease is mainly involved in mediating cell death processes, while caspases also have roles other than in apoptosis, e.g., caspase-1 (interleukin-1 beta convertase), which is involved in inflammatory processes (Abraham and Shaham, 2004; Lamkanfi et al., 2007). In the T class, small GTPases family (IPR001806), an independent superfamily within the larger class of regulatory GTP hydrolases (Bourne et al., 1990), was observed in *Fonsecaea* siblings ARF type (IPR02456), SAR1 type (IPR006687), ARF/SAR1 type (IPR006689) and Small GTPase, Ras type (IPR020849).

Within class O, post-translational modification protein turnover chaperones are involved in folding, maintenance, intracellular transport and degradation of proteins as well as in facilitating cell signaling. Many heat shock protein (Hsp) families have been identified in this study, such as Hsp20-like chaperone (IPR008979), Hsp40/DnaJ peptide-binding (IPR008971), Heat shock protein Hsp90, N-terminal (IPR020575), Heat shock protein Hsp90 family (IPR001404), Heat shock protein Hsp90, conserved site (IPR019805).

Likewise, using the functional annotation based on Gene Ontology (GO), a total of 7,392 genes were assigned (Figure S1). Of these genes, 409 were redundantly assigned into Cellular Component Ontology, 4,752 into Molecular Function Ontology and 2,231 into Biological Process Ontology. Most of the genes were annotated to binding (434) and oxyreductase (277) and methyltransferase (187) activity in the Molecular Function Ontology. In the Biological Process the overrepresented functions were metabolic processes (495), transport (239), and biosynthetic processes (195). The less represented ontology classes were membrane (87), protein complex (69) and chromosome (32) in Cellular Component Ontology.

Judging from these analyses, the presence and abundance of these functional domains could be related to the ecology of this agents, i.e., Siderophore iron transporter (GO:0015892, IPR010573) recovered by cells either by the reductive system or by specific transporters able to internalize the siderophore-iron complex (Itoh et al., 2000) and the Quinoprotein amine dehydrogenase (IPR011044), which is related to extremotolerance, this domain has been shown to interact with series of metal ions in anhydrous organic media (Matthews and Sunde, 2002).

In addition, GO enrichment analyses were used to determine the functional characteristics in the studied species (Table S4). It was observed that *F. erecta* presents a mechanism to regulate the use of nitrogen (GO: 0006808), a feature that may favor its survival in plants. Likewise, *F. erecta* and *C. yegresii* shared GO enrichments related to degradation of phenolic compounds such as L-phenylalanine catabolic process (GO: 0006559), tyrosine catabolic process (GO: 0006572) and homogentisate 1,2-dioxygenase activity (GO: 0004411). On the other hand, *C. carrionii* and *F. pedrosoi* exhibited an overrepresentation of the following GO terms: cellular response to iron ion starvation (GO: 0010106), siderophore biosynthetic process (GO: 0019290) and ferric triacetylfusarinine C transport (GO: 0015686) that are involved in iron metabolism.

### Species-specific genes

The gene families deduced by KOG annotations for each *Fonsecaea* species studied were presented in the Figure 5A. Considering the specific genes observed in each species (Figure 5B), the plant-associated *F. erecta* presented the largest number of genes related to General function prediction and Secondary metabolites biosynthesis transport and catabolism. The species had a Leucine-rich repeat variant (IPR004830, PF13855) and Universal stress protein signature (USP) (PF00582) domains. Moreover, the family fungal Fucose-specific lectin (IPR012475) and the domain Jacalin-type lectin domain profile (IPR001229) involved in metabolism of lectins also were found in this specie (Table S3). Zinc finger domains (Znf) are shared by *Fonsecaea* sibling, but some domains are present in specific proteins to some species (Table S3). The Znf are relatively small protein motifs, which contain multiple finger-like protrusions that make tandem contacts with their target molecules. There are many superfamilies of Znf motifs, varying in both sequence and structure (Matthews and Sunde, 2002).

### Virulence-related genes

Using PHI (Pathogen Host Interactions data base) (Winnenburg et al., 2007), genes of species belonging to the bantiana-and carrionii-clades were classified into categories considering virulence and pathogenicity. In total 3,865 genes were divided into 3 categories: Lethal 2,801 genes, Increased virulence (hypervirulence) 1,013 genes and Effector (plant avirulence determinant) 51 genes (Table S5). According to this analysis, 3,614 genes were present in all species studied that includes different transcription factors. On the other hand, 251 genes were limited to one or more species such as *Xeg1* observed in *F. erecta* and *Gr-VAP* gene in *C. yegresii* and *F. erecta*. In the class hypervirulence, all the analyzed species share the transcription factor *Amr1*, a gene described in *Alternaria brassicicola* this is an obligate plant pathogen as an inducer of melanin biosynthesis and associated with pathogenesis, suggesting that it is an important gene for the species to be a competitive saprophytic and also as a parasite (Cho et al., 2012). Moreover, the *RpfF* gene involved in pathogenesis of opportunistic pathogens (Suppiger et al., 2016) was observed in *F. multimorphosa, F. pedrosoi, F. monophora*, and *F. nubica* from the bantiana-clade and it was absent in the species from carrionii-clade (Table 3).

**Table 3:** *Fonsecaea* and *Cladophialophora* specific genes annotated in the PHI base.

**Species**	**Genes**	**Accession**	**Function in pathogenicity**
*F. erecta*	*Xeg1*	OAP56881.1	Triggered defense responses including cell death Ma et al., 2015
*F. erecta*	*GzHOMEL040*	OAP56314.1	Transcription factor Son et al., 2011
*F. erecta*	*GzZC062*	OAP64706.1	Transcription factor Son et al., 2011
*F. nubica*	*GzC2H089*	OAL38924.1	Transcription factor Son et al., 2011
*F. pedrosoi*	*RSc1356*	XP013280599.1	Gene effector in plant infection Pensec et al., 2015
*F. monophora*	*RSc1356*	OAG34226.1	
*F. nubica*	*RSc1356*	OAL31073.1	
F. multimorphosa^*^	*TcGALE*	OAL25194.1	Responsible for conidiation and mycelial development El-Ganiny et al., 2010
F. multimorphosa^**^	*COS1*	XP_016633043.1	Function as a transcriptional regulator controlling genes responsible for conidiation Zhou et al., 2009
*F. erecta*	*Gr-Vap1*	OAP625883.1	Defense-related programmed cell death in plant cells Lozano-Torres et al., 2012
*C. yegresii*	*Gr-Vap1*	XP_007761267.1	
*F. monophora*	*RpFf*	OAG36832.1	Regulation of biofilm formation, colony morphology, proteolytic activity, and virulence Suppiger et al., 2016
*F. pedrosoi*	*RpFf*	XP_013286584.1	
*F. nubica*	*RpFf*	OAL34548.1	
F. multimorphosa^*^	*RpFf*	OAL19303.1	
F. multimorphosa^**^	*RpFf*	XP_016628078.1	
*C. yegresii*	*AMR1*	XP_007752625.1	Inducer of melanin biosynthesis Cho et al., 2012
*C. carrionii*	*AMR1*	KIW73572.1	
*F. monophora*	*AMR1*	OAG33974.1	
*F. pedrosoi*	*AMR1*	XP_013282497.1	
*F. nubica*	*AMR1*	OAL32231.1	
F. multimorphosa^*^	*AMR1*	OAL18751.1	
F. multimorphosa^**^	*AMR1*	XP_016627577.1	
*F. erecta*	*AMR1*	OAP58408.1	
*C. immunda*	*AMR1*	XP_016244825.1	
*C. bantiana*	*AMR1*	XP_016622751.1	
*C. psammophila*	*AMR1*	XP_007742386.1	

* *CBS 980.69 isolated form brain disseminated infection in cat*.

** *CBS 102226 environmental isolate*.

### Fungal lifestyles expressed in peptidases and carbohydrate-active enzymes

**Figure 6:**
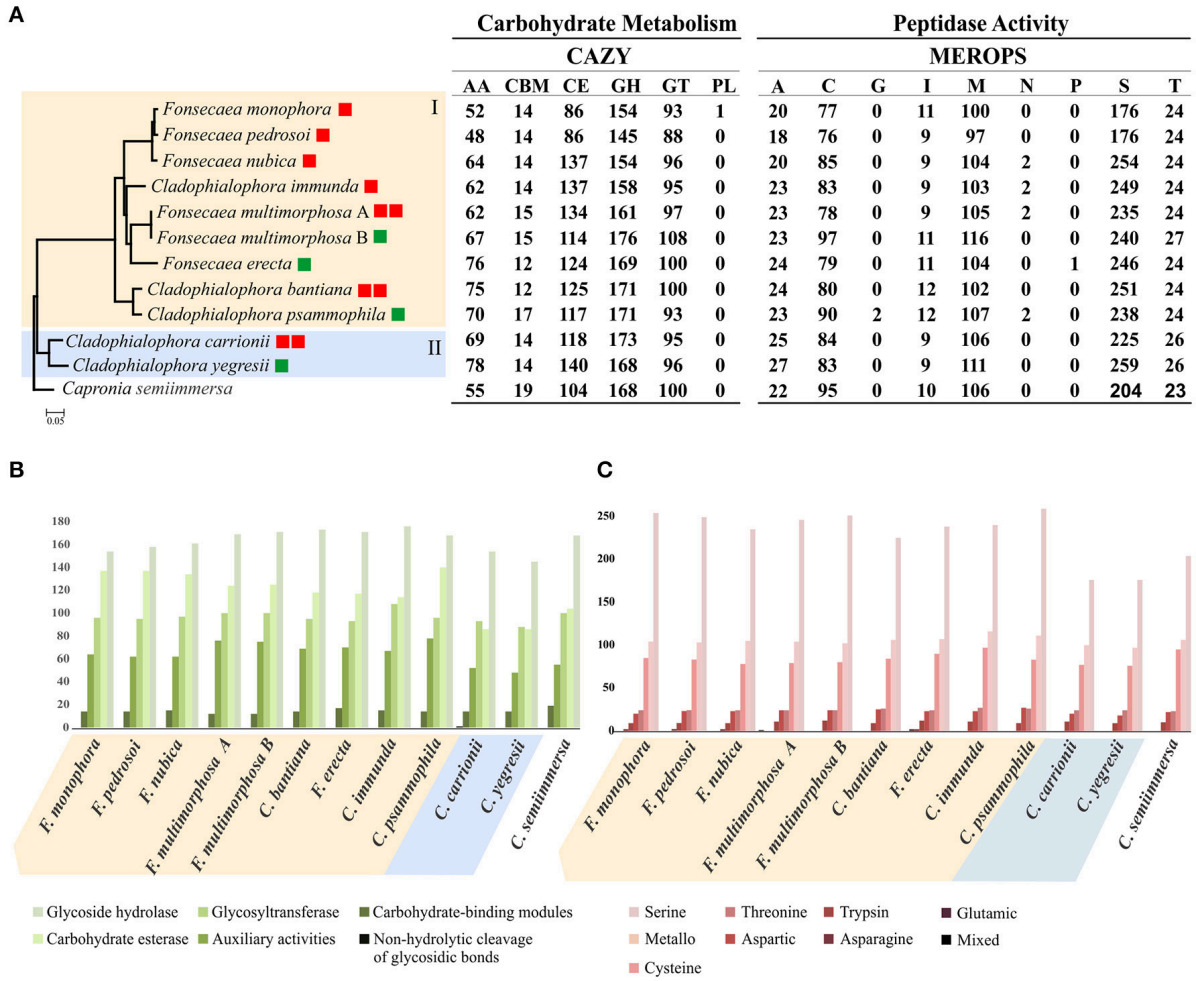
Analysis of carbohydrate and peptidase metabolism content in bantiana-and carrionii-clades. **(A)** Phylogenomic tree of bantiana- (I) and carrionii-clades (II) *Fonsecaea multimorphosa* **(A)** CBS 980.69. *Fonsecaea multimorphosa*. **(B)** CBS 102.226. All nodes on the phylogeny were supported by bootstrap values of 100% and the letters indicate ancestors. Species names followed by boxes: in green environmental strains, in red agents of chromoblastomycosis and cerebral infection. The ratio of MEROPS enzymes to CAZY enzymes for each genome is shown in the last column. **(B)** CAZY annotation: categories include AA (auxiliary activities), CBM (carbohydrate-binding modules), CE (carbohydrate esterase), GH (glycoside hydrolase), GT (glycosyltransferase) and PL (non-hydrolytic cleavage of glycosidic bonds). **(C)** MEROPS annotation: categories include A (aspartic), C (cysteine), G (glutamic), I (trypsin), M (metallo), N (asparagine), P (mixed), S (serine), and T (threonine).

The bantiana-and carrionii-clades were annotated with carbohydrate-active enzymes (CAZymes) database resulting in 5,058 genes encoding putative CAZymes, comprising 723 auxiliary activities (AA), 155 carbohydrate binding module (CBM), 1,318 carbohydrate esterases (CE), 1,800 glycoside hydrolases (GH), 1,061 glycosyl transferases (GT) and 1 polysaccharide lyase (Figure 6). The phylogenomic tree showing the carbohydrate and peptidase metabolism content in bantiana-and carrionii-clades was presented in the Figure 6A.

In both clades CAZymes associated with degradation of polysaccharides such as chitin, hemicellulose, glucans and pectin were the largest enzyme families. The number of glycoside hydrolase 43 (GH43) enzyme family related to pectin and hemicellulose degradation was higher in *F. erecta* than the other species (Table S6). In addition, in the bantiana-clade the activity carbohydrate esterase (CE) was higher than in the carrionii-clade, while no polysaccharide lyase (PL) was identified in *Fonsecaea* sibling genomes, only one in *C. carrionii* (Figure 6B).

Peptidase-encoding genes were predicted using the MEROPS database (Rawlings et al., 2015) (Table S7). The bantiana- and carrionii-clades were predicted to produce a wide repertoire of different endo- and exopeptidases. We identified for both clades 5,266 peptidases, of which the largest families were serine peptidases (2,549), metallo peptidases (1,155) and cysteine peptidases (912), wherein peptidase family S1 containing Serine endopeptidase was higher in the bantiana-clade (Figure 6C). The relation between CAZY and MEROPS) in bantiana- and carrionii-clades is shown in Figure 6 (family-specific classification shown in Tables S6, S7). The species in bantiana- and carrionii-clades showed similarity in gene contents, sharing different carbohydrate metabolism and peptidase genes (*p* = 0.00398; Mann-Whitney U test), suggesting that these fungi are able to degrade plant and animal substrates.

### Protein family expansion and contraction

**Figure 7:**
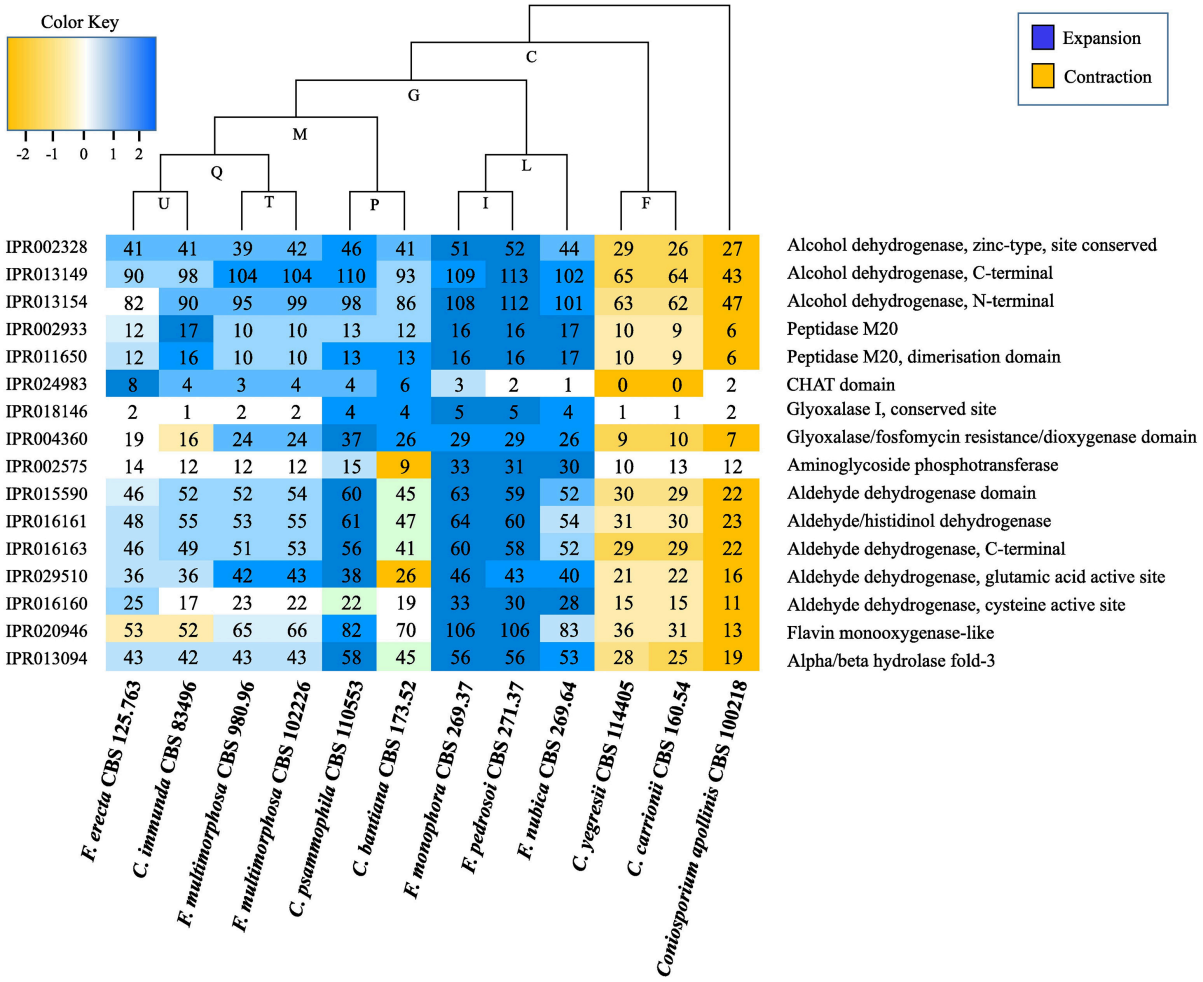
Interpro domains in *Fonsecaea* siblings. The letters indicate values for ancestry. The species are organized in phylogenetic order.

Protein domain expansions and contractions were inferred from the abundance of protein domains predicted by InterProScan searches and statistically tested by CAFE v3.0 (De Bie et al., 2006) (Table S8); letters indicating values for ancestors are shown in Figure 7 and Table S8. The adaptive functions of organism gene contents were evaluated by expansion and contraction of the protein family in *Fonsecaea* siblings in the bantiana-clade compared with species from the carrionii-clade. Several domains were expanded in the bantiana-clade, while in the carrionii-clade there is contraction demonstrating that different clades related to chromoblastomycosis are evolved in different directions due to ecological preferences (Figure 7).

Many domains and sites (IPR015590, IPR016161, IPR016163, IPR029510, and IPR016160) related to superfamily aldehyde dehydrogenase (ALDH) and alcohol dehydrogenase zinc type conserved site (IPR002328) showed an expansion in the ancestor of *F. pedrosoi, F. monophora* and *F. nubica*. Similarly, expansions in *F. erecta* and *C. bantiana* were observed in the protein family peptidase M20 (IPR002933 and IPR011650) and in the CHAT domain (IPR024983) related to caspases (Figure 7, Table S8).

Moreover, two domains associated with the glyoxal pathway, the glyoxalase I (IPR018146) and the glyoxalase/fosfomycin resistance/dioxygenase (IPR004360) are expanded in the ancestor of *F. pedrosoi, F. monophora*, and *F. nubica*. This enzyme catalyzes the first step of the glyoxal pathway, and in addition to its role in detoxifying glyoxal it may have other roles in stress response (Kim et al., 1993; Yim et al., 2001). Further this ancestor showed expansion in the aminoglycoside phosphotransferase (IPR002575) domain (Figure 7, Table S8); this domain consists of bacterial antibiotic resistance proteins, which confers resistance to various aminoglycosides (Trower and Clark, 1990; Chow, 2000).

### Virulence of *Fonsecaea* siblings

**Figure 8:**
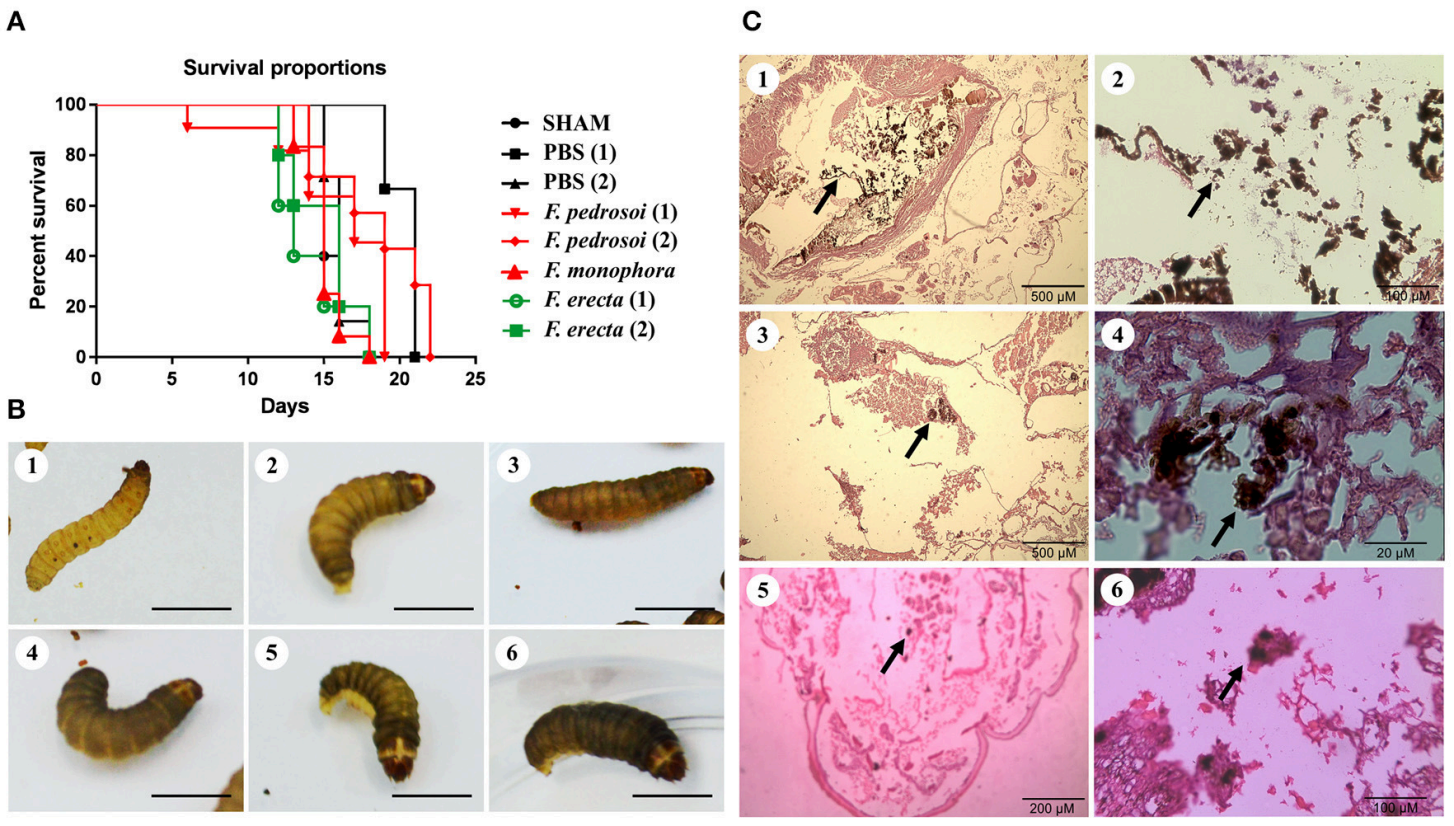
Virulence of *Fonsecaea* siblings using *Galleria mellonella* larvae. **(A)** Survival of *G. mellonella* larvae infected with *Fonsecaea* species. **(B)** Melanization of *G. mellonella* larvae infected with *Fonsecaea* species: (1, 2). Controls: SHAM and PBS; (3). *Fonsecaea erecta* CBS 125763; (4). *Fonsecaea monophora*; (5). *Fonsecaea pedrosoi* CBS 271.37; (6). *Fonsecaea pedrosoi* ATCC 46428. **(C)** Histology of infected tissue of *G. mellonella* with *Fonsecaea* species. The internal structures were fixed, embedded in paraffin and stained with PAS. Black arrows show hyphae spreading through the larva tissue. (1, 2). *Fonsecaea erecta* CBS 125763; (3, 4). *Fonsecaea pedrosoi* CBS 271.37*;* (5, 6). *Fonsecaea monophora* CBS 102248.

**Figure 9:**
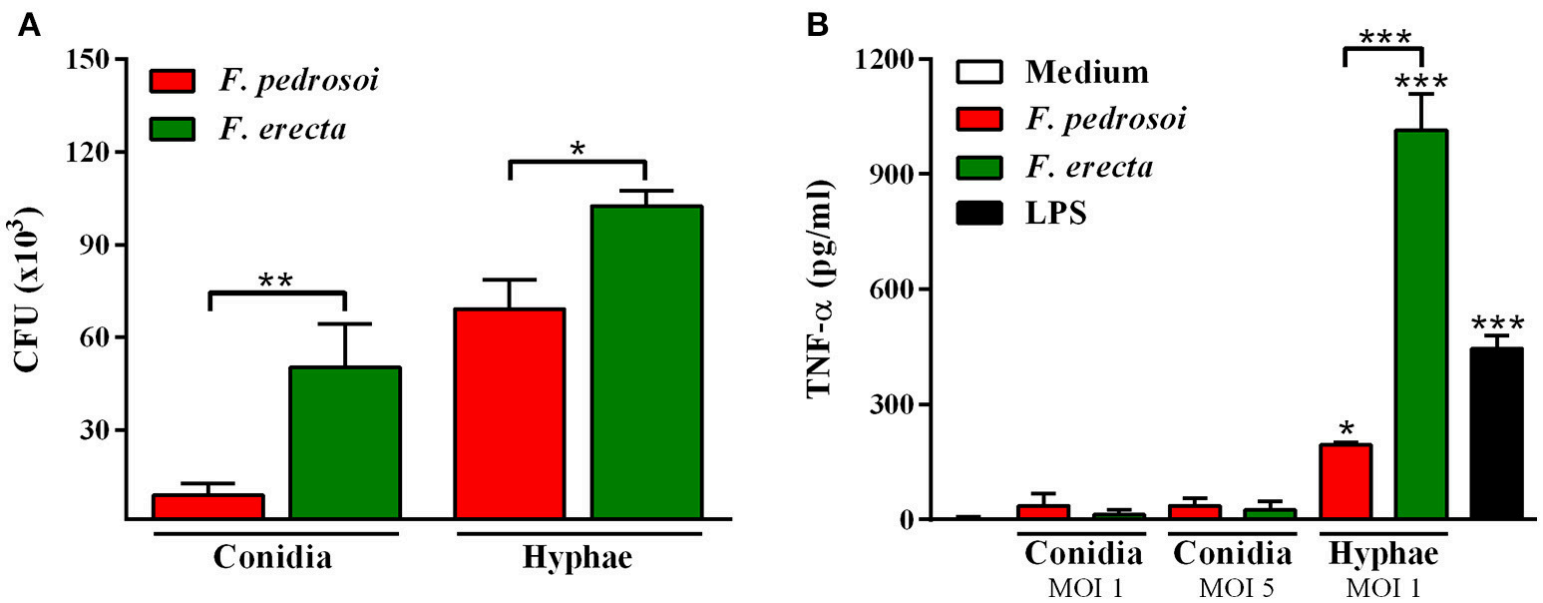
Fungal burden and production of pro-inflammatory cytokines of *F. pedrosoi* and *F. erecta*. **(A)** J774 murine macrophages (J774) were co-cultured with conidia or hyphal fragments, in the MOI 1 during 24 h. CFU data showed faster clearance of inoculated conidia from *F. pedrosoi* than *F. erecta*. **(B)** High levels of TNF-α were observed in macrophage co-culture with hypha, but not with conidia. Data were analyzed by one-way ANOVA with Tukey's *post-hoc* test. ^*^p < 0.05, ^**^p < 0.01, and ^***^p < 0.001 compared with *F. pedrosoi*; or compared with non-infected macrophages.

Survival tests using *Galleria mellonella* larvae as a model infected with *F. pedrosoi* ATCC 46428 and CBS 271.37^T^, *F. erecta* CBS 125763^T^, and *F. monophora* CBS 102248 showed a low larvae survival rate when compared to control groups. Larvae infected with *F. erecta* had a lower survival rate (Figure 8A). The infected larvae from the tested groups developed varying degrees of melanization, whereas the controls (PBS and SHAM) (Thomaz et al., 2013) did not present any melanization (Figure 8B1–6). Melanization could be observed from the first day post-inoculation in all tested groups. Histopathology (Figure 8C) showed the presence of pigmented nodules and granulomata in the tissue of infected larvae. Based on these results it was concluded that *F. erecta* CBS 125763^T^ is potentially able to infect animal hosts. In order to find out how *F. pedrosoi* and *F. erecta* conidia and hyphae trigger macrophage activation, microbicidal activity and TNF-α production was measured. *Fonsecaea erecta* hyphae are more resistant to macrophage destruction than *F. pedrosoi* after 24 h of co-culturing with macrophages *in vitro* (Figure 9). No difference was observed when macrophages were co-cultured with conidia from both *Fonsecaea* species (Figure 9A). Only hyphae from both *Fonsecaea* species were able to induce TNF-α secretion (Figure 9B).

**Figure 10:**
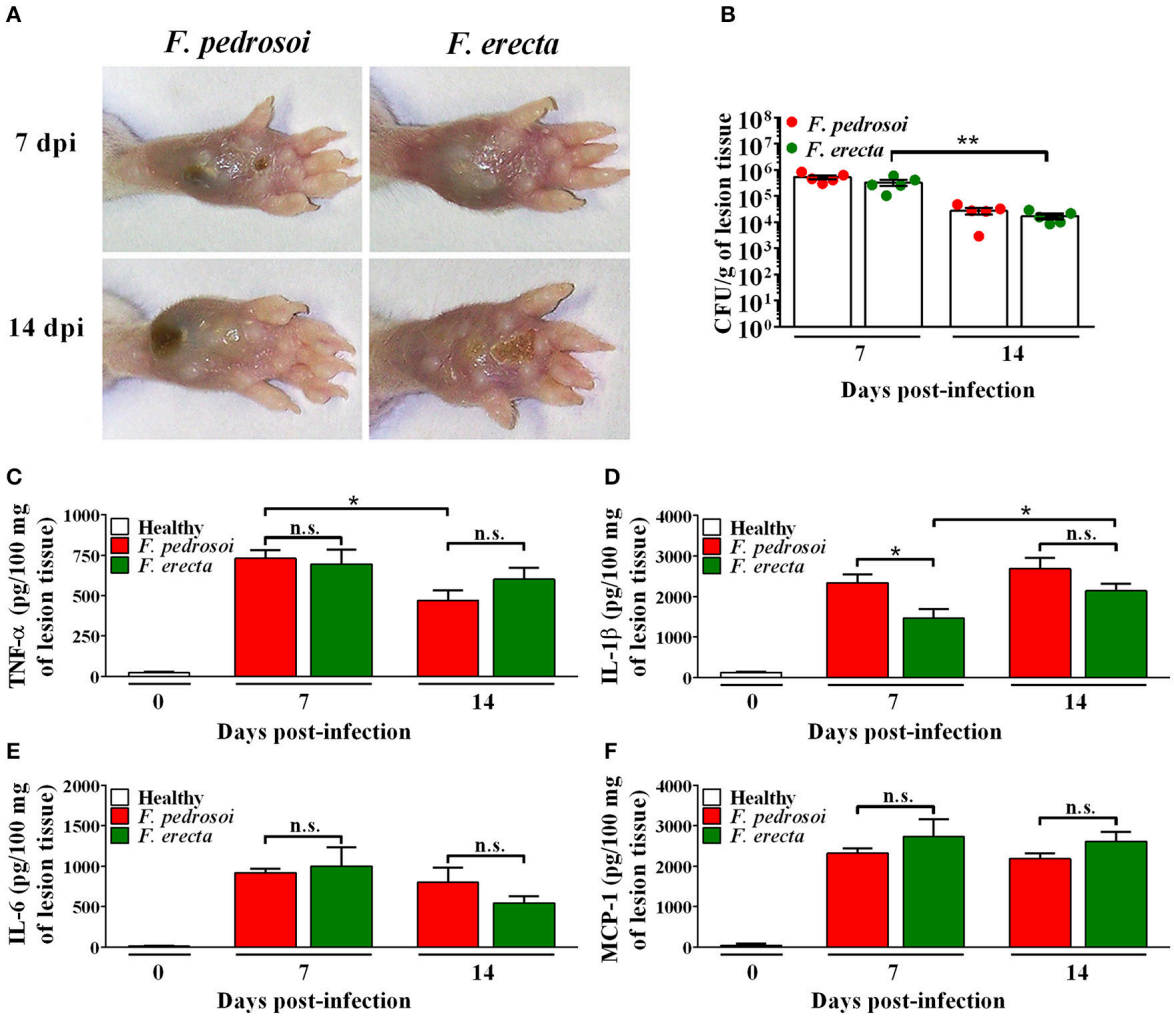
Virulence and immunostimulatory potential test of *Fonsecaea* sibling species using BALB/c mice as a model. **(A)** Macroscopic aspect of the disease. **(B)** CFU data showed a high tissue fungal burden of *F. erecta* which decline over the course of the infection. **(C–F)** At 7 and 14 days post-infection, high levels of TNF-α, IL-1β, IL-6 and MCP-1 were observed similarly in the footpad of mice infected with *F. pedrosoi* or *F. erecta*. Data were analyzed by two-way ANOVA with Tukey's post-hoc test. ^*^p < 0.05 and ^**^p < 0.01 between groups indicated by brackets; n.s.: not significantly.

*In vivo* assay using BALB/c mice as a model infected by pathogenic species *F. pedrosoi* and plant associated species *F. erecta* showed ulcerative and plaque type lesions. However, the *F. pedrosoi* infected mice showed lesions with dark plaque while *F. erecta* infected mice presented higher areas of edema (Figure 10A). All groups showed high levels of fungal burden at 7 dpi followed by reduction of viable fungi in the injured area (Figure 10B). The levels of cytokines were similar in both groups, except to IL-1β levels in *F. erecta* infected mice that increase lately when compared with *F. pedrosoi* infected mice (Figures 10C–F).

### Potential virulence genes related to human infection

An analysis of common genes related to the casual agents of disease showed 43 gene clusters shared by agents of systemic infection and 32 gene clusters related to (sub)cutaneous infection. Annotation of these clusters in *Fonsecaea* revealed 33 domains related to disseminated infection and 24 domains related to subcutaneous infection (Table 4).

**Table 4:** Prediction of virulence domains related to systemic and (sub)cutaneous infection.

**Systemic infection domains (Domain/ID access)**	**Function**	**(Sub)cutaneous infection (Domain/ID access)**	**Function**
AhpC/TSA Family/IPR000866	Antioxidant activity	Acyltransferase family/IPR002656	Transferase activity
Alpha/beta hydrolase fold/IPR000866	Catalytic activity	Alpha/beta hydrolase fold/IPR013094	Hydrolase activity
Amidase/IPR023631	Hydrolase activity	Amino acid permease/IPR004841	Transmembrane transport
Amidohydrolase Family/IPR006680	Hydrolase activity	Asp/Glu/Hydantoin racemase/IPR015942	Nitrogen compound metabolic process
C-terminal of 1-Cys peroxiredoxin/IPR019479	Peroxiredoxin activity	Cutinase/IPR000675	Hydrolase activity
DJ-1/PfpI Family/IPR002818	Glyoxalase	Flavin-binding monooxygenase-like/IPR020946	NADP binding
Unknown function (DUF1772)/IPR013901	Anthrone oxygenases	Fungal specific transcription factor/IPR007219	Zinc ion binding
Eukaryotic aspartyl protease/IPR033121	Proteolytic enzymes	Fungal Zn(2)-Cys(6) binuclear cluster/IPR001138	Zinc ion binding
FAD binding domain/IPR003953	Catalytic activity	Glutathione-dependent formaldehyde activating enzyme/IPR006913	Carbon-sulfur lyase activity
Flavin-binding monooxygenase-like/IPR020946	Metabolize xenobiotics	Glycosyl hydrolases family 16/IPR000757	Hydrolase activity
Flavin amine oxidoreductase/IPR002937	Oxidoreductase activity	LysM domain/IPR018392	Binding chitin
Fumarylacetoacetate (FAA) hydrolase Family/IPR011234	Catalytic activity	Major Facilitator Superfamily/IPR011701	Transmembrane transport
Fungal N-terminal of STAND proteins/IPR031348	Function is not known	Major intrinsic protein/IPR000425	Transporter activity
Fungal specific transcription factor/IPR007219	Zinc ion binding	Major royal jelly protein/IPR017996	Function is not known
Fungal specific transcription factor/IPR021858	Transcription factor	NAD dependent epimerase/dehydratase family/IPR001509	Catalytic activity
Fungal Zn(2)-Cys(6) binuclear cluster/IPR001138	Zinc ion binding	NADP oxidoreductase coenzyme F420-dependent/IPR028939	Catalytic activity
Glyoxalase/fosfomycin resistance protein/Dioxygenase superfamily/IPR004360	Catalytic activity	OTT_1508-like deaminase/IPR027796	Chromatin function
GMC oxidoreductase/IPR000172	Oxidoreductase activity	Oxidoreductase family, NAD-binding Rossmann fold/IPR000683	Oxidoreductase activity
GMC oxidoreductase/IPR007867	Oxidoreductase activity	Phosphotransferase enzyme family/IPR002575	Antibiotic resistance
Heterokaryon incompatibility protein (HET)/IPR010730	Preserve genetic individuality	Putative oxidoreductase C terminal/IPR013944	Oxidoreductase activity
Major Facilitator Superfamily/IPR011701	Transmembrane transport	Putative threonine/serine exporter/IPR010619	Catalytic activity
Multicopper oxidase/IPR001117	Oxidation-reduction	Pyrroline-5-carboxylate reductase dimerization/IPR029036	Dimerization domain
Multicopper oxidase/IPR011707	Copper ion binding	Sugar (and other) transporter/IPR005828	Transmembrane transporter
Multicopper oxidase/IPR011706	Oxidoreductase activity	Short chain dehydrogenase/IPR002347	Catalytic activity
NAD(P)H-binding/IPR016040	Catalytic activity	Threonine/Serine exporter, ThrE/IPR024528	Transmembrane transporter
NMT1-like Family/IPR011852	Protein receptors	—	—
Phenazine biosynthesis-like protein/IPR003719	Catalytic activity	—	—
Prion-inhibition and propagation IPR029498	Prion-inhibitory and propagation effect	—	—
Unknown function (DUF4243)/IPR025337	Function is not known	—	—
Sugar (and other) transporter/IPR005828	Transmembrane transporter activity	—	—
X-Pro dipeptidyl-peptidase C-terminal non-catalytic/IPR013736	Dipeptidyl-peptidase activity	—	—
X-Pro dipeptidyl-peptidase (S15 family)/IPR000383	Hydrolase activity	—	—

In the agents of systemic infection a high frequency was noted of the domains DJ-1/PfpI family (IPR002818) and glyoxalase/fosfomycin resistance protein/dioxygenase superfamily (IPR004360) related to glyoxal pathway, which have important roles in detoxifying glyoxals (Lee et al., 2013) and domains related to flavin proteins, such as monooxygenase-like flavin-binding (IPR020946) and flavin-containing amine oxidoreductase (IPR002937), and the family multicopper oxidases (MCOs) (IPR011706, IPR011707, and IPR001117) that includes laccases, ferroxidases, bilirubin oxidases and ascorbate oxidases (Hoegger et al., 2006).

The subcutaneous agents shared a number of domains related to enzymes and transporters (Table 4), such as major facilitator, sugar transporter-like (IPR005828) and major facilitator superfamily IPR011701), aggregating several families of transporters. Among them a siderophore transporter, RhtX/FptX family and the yellow-like family is a gene class characterized by the presence of a major royal jelly protein (MRJP) domain (Ferguson et al., 2011) with multiple physiological and development functions in insects (Drapeau et al., 2003), such as synthesis of melanin pigments and sex-specific reproductive maturation. A large variety of enzymes such as acyltransferases (IPR002656), hydrolases (IPR013094 and IPR000757), proteases, lipases, permeases (IPR004841) and dehydrogenases (IPR002347) were observed. Nevertheless, also domains related to invasion of the plant tissue were observed, such as cutinase (IPR000675) (Dickman et al., 1989) and LysM domain related to chitinase (IPR018392) (Gruber and Seidl-Seiboth, 2012).

## Discussion

The genus *Fonsecaea* is located in the Chaetothyriales, family Herpotrichiellaceae and contains species of environmental saprobes on plants or plant debris and pathogenic species, associated with subcutaneous and deep infections in human and animal hosts. The environmental species are consistently distinct from the *Fonsecaea* spp. commonly founded as agents of disease. The same phenomenon of environmental next to pathogenic species is known in *Cladophialophora* (Vicente et al., 2014).

Phylogenomic analysis shows that *Fonsecaea* is intermingled with some *Cladophialophora* species, while *Cladophialophora* agents of chromoblastomycosis are located in a separate carrionii-clade (Figure 3A). The invasive potential of black yeast-like fungi is known to differ significantly between species (Badali et al., 2008; Seyedmousavi et al., 2011). Both clades with agents of chromoblastomycosis also contain non-pathogenic representatives. Formation of muriform cells in host tissue is considered as the hallmark of the disease, but some environmental species are able to produce such cells *in vitro* (Badali et al., 2008).

Genomes of species of *Fonsecaea* under study are quite similar. An abundance of domains related to zinc-containing alcohol dehydrogenase (ADH) was observed (Figure 4), which have multiple biological functions such as detoxification. Similar results have been reported for other black yeasts (Teixeira et al., 2017), suggesting that tolerance of extreme and toxic environmental habitats is a mainstay in the ecology of black yeast-like fungi. Likewise, the superfamily ALDH represents enzymes that metabolize a wide variety of endogenous and exogenous aldehydes (Lindahl, 1992; Vasiliou et al., 2000). The expansion of ALDH in the ancestor of *Fonsecaea* (Figure 7) indicates metabolic plasticity explaining a dual ecological ability, surviving hostile environments as well as mammal host tissue. Also Flavin-containing monooxygenases (FMOs) play a role in a wide variety of processes such as the detoxification of drugs, biodegradation of aromatic compounds, siderophore biosynthesis and biosynthesis of antibiotics (van Berkel et al., 2006), which could thus be further key executors in processes related to extremotolerance.

Gene Ontology (GO) annotation indicated presence of siderophore iron transporters in the core genome of *Fonsecaea* (Figure S1). Most fungi are able to produce and utilize intracellular siderophores as an iron storage compound (Eisendle et al., 2006). In *Candida albicans* siderophore transporter-defective mutants were clearly compromised in invading keratinocyte layers suggesting that siderophore uptake is required to epithelial invasion and penetration (Leon-Sicairos et al., 2015). The clinical species *C. carrionii* and *F. pedrosoi* show gene enrichment in cellular response to siderophore biosynthetic process and ferric triacetylfusarinine C transport which might play a role in virulence of these agents, as these genes were not enriched in the environmental species (Table S4).

*Fonsecaea* siblings produce melanin via different pathways demonstrating that in these fungi the pathways are conserved (Chen et al., 2014; Teixeira et al., 2017). Melanin and carotenoids deposited in the cell walls are considered a putative virulence factor in established human pathogens such as *Histoplasma capsulatum, Sporothrix schenckii*, and *Cryptococcus neoformans*. Melanin is associated to the survival and competitive abilities of fungi in hostile environments (Nosanchuk et al., 2002; Mednick et al., 2005; Nosanchuk, 2005; Taborda et al., 2008) and also enhances tolerance of oxygenic burst in macrophages. Key enzymes involved in melanin biosynthetic pathways belong to the family Multicopper oxidases (MCOs) including laccases, ferroxidases, bilirubin, oxidases and ascorbate oxidases that catalyze the oxidation of a variety of substrates and mainly aromatic compounds (Hoegger et al., 2006). Laccases form the largest subgroup and are considered key enzymes of the DOPA melanin pathway (Walton et al., 2005). They are abundant in fungal genomes, related to their divergent physiological roles and differential regulation upon changing environmental conditions (Cañero and Roncero, 2008; Giardina et al., 2010). Teixeira et al. (2010) suggested that melanin pigments protect the fungus from the mammalian host's innate immune responses providing resistance to oxidizing agents and fungal cell death during phagocytosis. Melanin is important because has a role in the protection against antifungal drugs (van den Sande et al., 2007) and is significant in differentiation of the muriform cell, the invasive form of chromoblastomycosis.

The glyoxalase system consists of two consecutive enzymatic reactions (Glyoxalase I and II) with the terminal product D-lactate by metabolism of the physiological substrate methylglyoxal. The widespread distribution of glyoxalase in prokaryotic and eukaryotic cells suggests it fulfills a function of fundamental importance to life. Inhibition of the glyoxalase system leads to methylglyoxal accumulation to toxic levels. It has been implicated in control of cell growth and proliferation, and detoxification of methylglyoxal (Thornalley, 1993). Glyoxalase enzymes are modified during phagocytosis, and the enzymatic reaction has been implicated as a virulence factor for neutralization of the immune response during invasion (Gillespie, 1978, 1981). It has been reported in sugar-limited environments, the fungus relies on fatty acid metabolism for growth (Sexton and Howlett, 2006). Accumulation of lipid in thick-walled resting cells at the expense of sugars is a key mechanism in yeast-to-mold conversion in black yeasts (Oujezdsky et al., 1973). Glyoxalase might thus play a central role in the response to varying conditions. In *Fonsecaea* the pathway is expanded in the clinical species of the bantiana-clade (Figure 7) suggesting that the glyoxal pathway cycle might be required for virulence during invasion, in addition to its role in surviving extreme environmental conditions (Teixeira et al., 2017).

Caspases occur in the *Fonsecaea* core genome where they are synthesized as pro-proteins, possessing weak proteolytic activity (Madeo et al., 2002; Cheng et al., 2003; Abraham and Shaham, 2004) and also induce apoptosis enhancing pathogenesis (Douglas et al., 2015). Activation of apoptosis may lead to caspase-1 activation, providing a link between apoptosis and inflammation (Schumann et al., 1998; Lamkanfi et al., 2007). In the T class, a small GTPase family and an independent superfamily of GTP-binding proteins share enzymatic activity and play pivotal roles in cell division, protein synthesis and signaling (Paduch et al., 2001). Small GTPase—Ras type (IPR020849) is the most represented domain regulating cell growth, proliferation and differentiation (Table S1). The Ras family of GTPase proteins has been shown to control morphogenesis in many organisms, including pathogenic fungi such as *Cryptococcus neoformans* (RAS1) (Alspaugh et al., 2000), *Candida albicans* (CaRAS1 and CaRSR1) (Leberer et al., 2001) and *Aspergillus fumigatus* (rhbA) (Panepinto et al., 2003).

Heat shock proteins (Hsp) in the core genome (Table S1) are essential eukaryotic molecular chaperones, being first proteins that are up-regulated under conditions of elevated temperature (Lund, 2001). Especially, Hsp90 chaperones are unique in their ability to regulate a specific subset of cellular signaling proteins that have been implicated in disease, including intracellular protein kinases, steroid hormone receptors and growth factor receptors (Tamayo et al., 2013), which are likely mechanism of mammal infection where elevated temperature is an essential condition (Vicente et al., 2012).

The above discussed domains may explain the capacity to survive extreme conditions, of which living mammal tissue is one, but do not explain differences between species of the same clade. The significant predisposition observed in agents of chromoblastomycosis (Vicente et al., 2001, 2008, 2014) probably rests upon diversity enzymatic reactions. CAFE analysis of *Fonsecaea pedrosoi* and *Cladophialophora carrionii*, both causing this disease, evolve in opposite directions, as several domains expanded in the bantiana-clade appeared to be contracted in the carrionii-clade (Figure 7). This may demonstrate that members of different clades causing chromoblastomycosis have evolved in different directions due to clade-specific ecological preferences, or perhaps more likely that the displayed domains *in toto* reflect phylogeny rather than ecology.

The CAZy Database is a powerful reporter of fungal lifestyles once the fungi degrade an enormous functional and structural diversity of complex plant polysaccharides. Zhao et al. (2013) revealed that most fungi that lack PL and tend to lose CE8, CE11, GH6, GH73, GH80, and GH82 families are saprobes; these were also observed in bantiana- and carrionii-clades. A wide variety of extracellular peptidases is produced to degrade a gamut of environmental substrate complexes, indicative for a less specialized nutritional status (da Silva et al., 2006; Sriranganadane et al., 2010). Species of the bantiana-and carrionii-clades produce enzymes involved in plant cell wall pectin and hemicellulose degradation. Besides, in both clades a significant similarity was observed among gene content related to carbohydrate metabolism and peptidase (Figure 6). It suggests that these fungi are able to degrade plant and animal substrates demonstrating a duality in lifestyle that could enable Chaetothyriales pathogenic species to transfer from environmental niches to animal material. The similarity of carbohydrate-active and protein degrading enzymes associated to the occurrence of additional virulence factors, which may support the tolerance to extreme environmental niches of the fungus (Teixeira et al., 2017), suggests an opportunistic tendency of *Fonsecaea* sibling species.

Primary fungal pathogens attempt to disrupt host cell homeostasis while avoiding and/or suppressing host recognition. In opportunists these mechanisms are not sophisticated and probably have emerged due to flexibility in nutrient acquisition (Dickman and Figueired, 2011) and extremotolerance (Moreno et al., 2017). Prenafeta-Boldú et al. (2006) and Casadevall (2007) suggested that this unfocused virulence explains the “dual use” determinants in unexpected agents of disease. It is likely that this principle also holds true for most black yeast-like fungi. However, as some common agents of chromoblastomycosis seem to have a significant predilection for this disease and are rarely found in the environment (Vicente et al., 2014) a certain degree of pathogenic adaptation cannot be excluded.

The subcutaneous infections by *Fonsecaea* and *Cladophialophora* species frequently result from a trauma from environmental sources. The muriform cell, considered to be a chromoblastomycosis-specific tissue form in humans, has been observed in cactus thorns infected with *C. yegresii* (De Hoog et al., 2007) and also *in vitro* in several environmental species (Badali et al., 2008). The *RSc1356* effector is involved in plant infection (Pensec et al., 2015) and its presence in *Fonsecaea* might support this hypothesis, although plant- and human-pathogenicity are almost mutually exclusive (De Hoog et al., 2000). The use of nitrogen and degradation of phenolic compounds that are also enriched in environmental *F. erecta* and *C. yegresii* (Table S7) are more likely causes of opportunism. The class of protein lectins (Table S3), which is implicated in many essential cellular and molecular recognition processes (Varrot et al., 2013) was present *F. erecta* isolated from plant material. De Hoog et al. (2004) stated that pathology on humans is coincidental, humans not being the primary hosts of these fungi.

The above hypothesis is partially supported by results of virulence testing using *G. mellonella* larvae as a model. Larvae infected by environmental *F. erecta* had a lower survival than those infected by clinical strains of *F. pedrosoi* (ATCC 46428 and CBS 271.37^T^) and *F. monophora* (CBS 102248). In addition, *F. erecta* hyphae induced high levels of TNF-α (Figure 9), contributing to macrophage activation after phagocytosis. Macrophages activated by TNF-α increase their ability to control intracellular fungal growth, stimulate recruitment of inflammatory cells and stimulate the formation and maintenance of granulomata (Algood et al., 2005; Juhász et al., 2013; Gyurkovska and Ivanovska, 2016). Although *F. erecta* hyphae induced high levels of TNF-α, hyphal death was not observed, suggesting a higher level of extremotolerance. The higher virulence of strictly environmental *Fonsecaea* species does however not explain which the species which are commonly found on human hosts show lower virulence in the *Galleria* model.

Furthermore, based on PHI database the genes classified as lethal are mostly transcription factors (TFs) that orchestrate gene expression which determines life and functionality of the cell (Shelest, 2008) by controlling cellular signaling pathways and thus are key mediators of cellular function of fungi (Shelest, 2008; Wang et al., 2011).

The high number of domains related to enzymes and transporters reveals important mechanisms of nutrient acquisition and extremotolerance, constituting a genomic machinery that allows hydrolysis of recalcitrant components present in plant debris and suggests multiple survival strategies including mammal infection. Explanation of the observed differences in prevalence in the human host between closely related species appeared nevertheless impossible, as illustrated by the lower *Galleria* survival rate after infection with the non-pathogenic *F. erecta*. The large number of proteins (Figure 4, Table 4) with unknown function demands further investigation of these genes and their potential role in survival; small but crucial differences between these closely related fungi may have been concealed in the present set of proteins studied. Despite the close relationship in classical marker genes, *Fonsecaea* species are surprisingly different in their mitochondrial genomes, which in most fungi are highly conserved (Torriani et al., 2014; Jelen et al., 2016). Differences in routes of transmission allowing passage of properties acquired in the host to a next generation and thus, allowing evolutionary adaptation (Queiroz-Telles et al., 2017) are not easily revealed.

## Author contributions

Conceived and designed the experiments: VV, VW, LM, RR, ALB, SA, ES, SDH. Generation sequence data: HF, MZTS, VB, EB. Performed the experiments: GF, ALB, RdC, SA, SDS, MMFdN. Analyzed the data: VV, VW, AB, LM, FC, RR, AL. Contributed reagents/materials/analysis tools: RR, VV, FP, ES. Contributed to preparing the manuscript and revising it critically: VV, VW, AB, FC, AL, RG. Annotation and analysis of data; preparation, creation and/or presentation of the tables; graphics and figures: VW, AB, LM, FC, RR, AL, RG. Strains offered and/or Substantial contributions to the work FQ, ALB, SA, MMFN, SDH. Conceived and revised paper: JS, MT, MSF, MS, DA, MJN, VV, ES, SDH. Conception and design of the work and writing the manuscript. VV, VW, AB, ES, SDH.

### Conflict of interest statement

The authors declare that the research was conducted in the absence of any commercial or financial relationships that could be construed as a potential conflict of interest.
