# Supplementary figures and images for: Comparative Genomics of Sibling Species of Fonsecaea Associated with Human Chromoblastomycosis
Source: Front Microbiol. 2017 Oct 9;8:1924. doi: 10.3389/fmicb.2017.01924 (PMC5640708; doi:10.3389/fmicb.2017.01924)

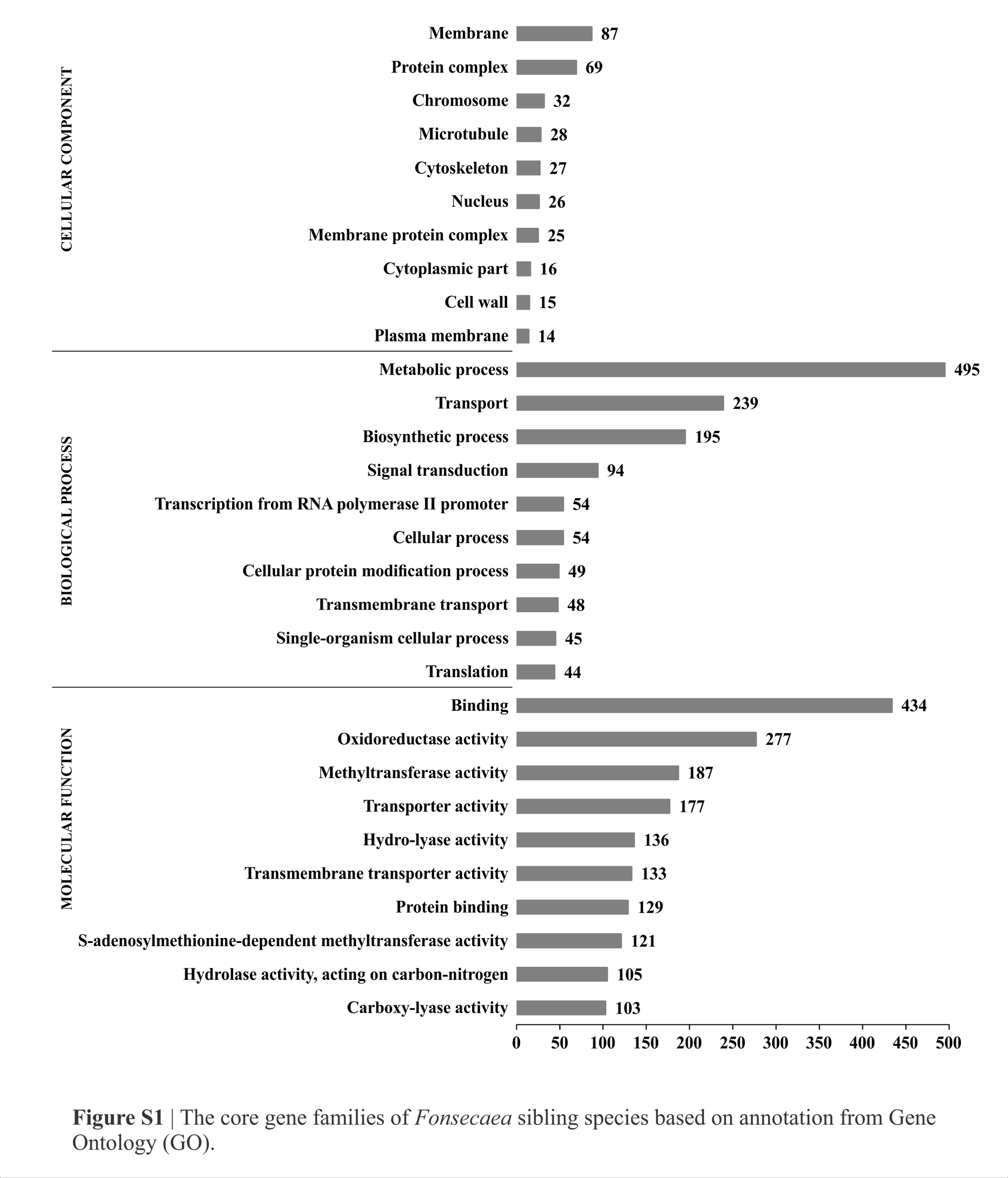

Supplement: Supplementary file 9 [file Image1.tif]
